# A Dynamical Generative Model of Social Interactions

**DOI:** 10.3389/fnbot.2021.648527

**Published:** 2021-06-09

**Authors:** Alessandro Salatiello, Mohammad Hovaidi-Ardestani, Martin A. Giese

**Affiliations:** Section for Computational Sensomotorics, Department of Cognitive Neurology, Centre for Integrative Neuroscience, Hertie Institute for Clinical Brain Research, University Clinic Tübingen, Tübingen, Germany

**Keywords:** social interactions, generative model, motion cues, social perception, social inference

## Abstract

The ability to make accurate social inferences makes humans able to navigate and act in their social environment effortlessly. Converging evidence shows that motion is one of the most informative cues in shaping the perception of social interactions. However, the scarcity of parameterized generative models for the generation of highly-controlled stimuli has slowed down both the identification of the most critical motion features and the understanding of the computational mechanisms underlying their extraction and processing from rich visual inputs. In this work, we introduce a novel generative model for the automatic generation of an arbitrarily large number of videos of socially interacting agents for comprehensive studies of social perception. The proposed framework, validated with three psychophysical experiments, allows generating as many as 15 distinct interaction classes. The model builds on classical dynamical system models of biological navigation and is able to generate visual stimuli that are parametrically controlled and representative of a heterogeneous set of social interaction classes. The proposed method represents thus an important tool for experiments aimed at unveiling the computational mechanisms mediating the perception of social interactions. The ability to generate highly-controlled stimuli makes the model valuable not only to conduct behavioral and neuroimaging studies, but also to develop and validate neural models of social inference, and machine vision systems for the automatic recognition of social interactions. In fact, contrasting human and model responses to a heterogeneous set of highly-controlled stimuli can help to identify critical computational steps in the processing of social interaction stimuli.

## 1. Introduction

Human and non-human primates are able to recognize the social interactions taking place in their environment quickly and effortlessly: with a few glances out of the window, we can easily understand whether two people are following each other, avoiding each other, fighting, or are engaging in some other form of social behavior. Notably, such interactive behaviors can be recognized even when the available visual information is poor: for example, when the scene we are watching is unfolding behind the leaves of a tree, at a considerable distance from us, or in a low-resolution video. In some of these situations, critical visual cues such as facial expressions might be completely occluded, yet our ability to make social inference is largely unaffected. Such perceptual ability is instrumental in allowing us to move in our social environment and flexibly interact with it, while abiding by the social norms (Troje et al., 2013). Therefore, it constitutes an important social skill that is worth characterizing and modeling also for the development of social robots.

Understanding the neural mechanisms underlying the inference of animacy and social interactions from visual inputs is a long-standing research challenge (Heider and Simmel, 1944; Michotte, 1946; Scholl and Tremoulet, 2000; Troje et al., 2013). Recent work has started identifying some of the responsible neural circuits (Castelli et al., 2000; Isik et al., 2017; Sliwa and Freiwald, 2017; Walbrin et al., 2018; Freiwald, 2020). Even though the detailed computational mechanisms mediating the formation of social percepts from visual inputs remain largely unknown, converging evidence has shown that the observation of biological motion alone is enough for humans to make accurate social inferences (e.g., Heider and Simmel, 1944; Tremoulet and Feldman, 2000; McAleer and Pollick, 2008; Roether et al., 2009). For example, Heider and Simmel (1944) demonstrated that humans can reliably decode animacy and social interactions from strongly impoverished stimuli consisting of simple geometrical figures moving around in the two-dimensional plane. Remarkably, despite their highly abstract nature, the visual stimuli used in this study were perceived as *alive* and sometimes even *anthropomorphic*: the agents were often considered as endowed with intentions, emotions, and even personality traits.

Several subsequent studies (e.g., Oatley and Yuill, 1985; Rimé et al., 1985; Springer et al., 1996; Castelli et al., 2000, 2002) replicated these findings using similar stimuli and showed that the inference of social interactions from impoverished stimuli is a cross-cultural phenomenon (Rimé et al., 1985) that is present even in 5-year-old preschoolers (Springer et al., 1996). Taken together, these findings support the view that the perception of animacy and social interactions might rely on some innate and automatic processing of low-level kinematic features present in the visual inputs, rather than on higher-level cognitive processing (Scholl and Gao, 2013).

The identification of the most critical visual features that shape these social percepts has also received great attention (Tremoulet and Feldman, 2000, 2006). For example, influential work suggested that these percepts are mediated by the detection of apparent violations of the principle of conservation of energy (Dittrich and Lea, 1994; Gelman et al., 1995; Csibra, 2008; Kaduk et al., 2013). Later research proved that also agent's orientation, velocity, and acceleration play a major role (Szego and Rutherford, 2008; Träuble et al., 2014). At the same time, neuroimaging work has shed light on some of the brain regions mediating these phenomena: the right posterior superior temporal sulcus (pSTS—Isik et al., 2017; Walbrin et al., 2018), the medial prefrontal cortex (mPFC—Castelli et al., 2000; Sliwa and Freiwald, 2017), and the right temporoparietal junction (TPJ—Castelli et al., 2000; Saxe and Kanwisher, 2003) are among the brain regions most frequently reported as being involved in the perception of social interaction. Interestingly, Schultz and Bülthoff (2019), recently identified another region—the right intraparietal sulcus (IPS)—that seems to be exclusively engaged during the perception of animacy.

Clearly, the success of both behavioral and neuroimaging social perception studies is tightly linked to the ability to finely control the visual stimuli that participants are exposed to. Specifically, such stimuli should ideally be generated through a process that allows complete parametric control, the creation of a high number of replicates with sufficient variety, and the gradual reduction of complexity. *Parametric control* (e.g., over agents' speed) facilitates the identification of brain regions and individual neurons whose activation covaries with the kinematic features of agents' behavior. *Variety* in classes of social interaction allows the characterization of the class-specific and general response properties of such brain regions. *Numerosity* allows averaging out response properties that are independent of social interaction processing. Finally, the ability to control stimulus complexity allows the generation of *impoverished stimuli* that are fundamental to minimize the impact of confounding factors, inevitably present, for example, in real videos. Similarly, such properties are also desirable when designing and validating neural and mechanistic models of human social perceptions: contrasting human and model responses to a variety of highly controlled stimuli can help discriminate between the computational mechanisms that the models capture well from those that need further refinement. This is especially critical for state-of-the-art deep learning models (e.g., Yamins et al., 2014), which can easily have millions of parameters and be prone to over-fitting.

Currently, no well-established method can generate visual stimuli for the analysis of social perception that satisfy all of the above conditions. Because of this, researchers often have to resort to time-consuming and class-specific, heuristic procedures. A creative approach to this problem has been the one adopted by Gordon and Roemmele (2014), where the task of generating videos was assigned to a set of participants—who were asked to create their own videos of socially interacting geometrical shapes, and to label them accordingly. However, typically, researches use visual stimuli where agents' trajectories are hand-crafted or hard-coded (e.g., Heider and Simmel, 1944; Oatley and Yuill, 1985; Rimé et al., 1985; Springer et al., 1996; Castelli et al., 2000, 2002; Baker et al., 2009; Gao et al., 2009, 2010; Kaduk et al., 2013; Träuble et al., 2014; Isik et al., 2017; van Buren et al., 2017; Walbrin et al., 2018), based on rules (e.g., Kerr and Cohen, 2010; Pantelis et al., 2014), or derived from real videos (e.g., McAleer and Pollick, 2008; McAleer et al., 2011; Thurman and Lu, 2014; Sliwa and Freiwald, 2017; Shu et al., 2018). All of these approaches suffer from significant limitations. Hand-crafted trajectories need to be generated *de novo* for each experimental condition and are not easily amenable to parametric control. Likewise, the extraction of trajectories from real videos also comes with its burdens: real videos need to be recorded, labeled, and heavily processed to remove unwanted background information. Rule-based approaches offer an interesting alternative. However, it is generally difficult to define natural classes of social interactions using rules akin to those used in Kerr and Cohen (2010) and Pantelis et al. (2014). Recent work (Schultz and Bülthoff, 2019; Shu et al., 2019, 2020) has generated visual stimuli using model-based methods; however, these models can only generate limited and generic classes of social interaction (namely, cooperative and obstructive behaviors). Finally, specialized literature on the collective behavior of humans and animals has produced a wealth of influential models (Blackwell, 1997; Paris et al., 2007; Luo et al., 2008; Russell et al., 2017); however, such models can also typically account only for simple behaviors (e.g., feeding, resting, and traveling) and for basic interactions (e.g., avoidance and following).

To overcome the limitations of the above methods, in this work, we introduce a dynamical generative model of social interactions. In stark contrast to previous work, our model is able to automatically generate an arbitrary number of parameterized motion trajectories to animate virtual agents with 15 distinct interactive motion styles; the modeled trajectories include the six fundamental interaction categories frequently used in psychophysical experiments (i.e., *Chasing, Fighting, Flirting, Following, Guarding*, and *Playing*—Blythe et al. 1999; Barrett et al. 2005; McAleer and Pollick 2008) and nine relevant others. The model controls *speed*, and *motion direction*, arguably the two most critical determinants of social interaction perception (Tremoulet and Feldman, 2000; Szego and Rutherford, 2008; Träuble et al., 2014). Finally, we validated the model with three psychophysical experiments, which demonstrate that participants are able to consistently attribute the intended interaction classes to the animations generated with our model.

The rest of the paper is organized as follows. In section 2, we describe the generative model and the experiments we conducted to validate it. Next, in section 3, we summarize the experimental results. Finally, in section 4, we (1) explain how our results validate the developed model, (2) explain how the model compares to related work, and (3) discuss the main limitations of our model and future directions.

## 2. Methods

### 2.1. Related Modeling Work

The generative model we introduce in this work builds on classical models of biological and robotic navigation. In the classical work by Reichardt and Poggio (1976), the authors proposed a dynamical model to describe the navigation behavior of flies intent on chasing moving targets as part of their mating behavior. The core idea was to consider the moving targets as *attractors* of the dynamical system describing the flies' trajectories. Subsequently, Schöner and Dose (1992) and Schöner et al. (1995) used a similar approach to develop a biomimetic control system for the navigation of autonomous robots. Critically, such a system was also able to deal with the presence of obstacles in the environment, which were modeled as *repellors*. Extending this system, Fajen and Warren (2003) built a model of human navigation that was able to closely capture the trajectories described by their participants as they walked naturally toward targets while avoiding obstacles on their way. Specifically, this model was able to describe the dynamics of the participants' average heading direction very accurately; however, their speed was roughly approximated as constant.

Alternative approaches can characterize richer navigation behaviors by jointly modeling both heading direction and speed dynamics. This idea was successfully used to control the motion of both autonomous vehicles (Bicho and Schöner, 1997; Bicho et al., 2000) and robotic arms (Reimann et al., 2011). Similar approaches have also been used in computer graphics to model the navigation of articulated agents (Mukovskiy et al., 2013).

### 2.2. The Generative Model

To model the interactive behavior of two virtual agents, we define, for each agent *i*, a dynamical system of two nonlinear differential equations. Specifically, the equations describe the dynamics of the agent's heading direction ϕ_*i*_(*t*) and instantaneous propagation speed *s*_*i*_(*t*).

The heading direction dynamics, derived from Fajen and Warren (2003), are defined by:

(1) $$\begin{array}{l} \ddot{\phi_{i}}\left(t\right)=-b\dot{\phi_{i}}\left(t\right)+A\left(\phi_{i}\left(t\right),\psi_{i}^{g}\left(t\right)\right)+R\left(\phi_{i}\left(t\right),\psi_{i}^{o}\left(t\right)\right) \end{array}$$

In this equation, $A\left(\phi_{i}\left(t\right),\psi_{i}^{g}\left(t\right)\right)$ defines the *attraction* of agent *i* to the goal *g* located along the direction $\psi_{i}^{g}\left(t\right)$, at a distance $d_{i}^{g}\left(t\right)$ from it. Similarly, $R\left(\phi_{i}\left(t\right),\psi_{i}^{o}\left(t\right)\right)$ defines the *repulsion* of agent *i* for the obstacles $o={\left[o_{1},o_{2},...,o_{N_{obst}}\right]}^{T}$ located along the directions $\psi_{i}^{o}\left(t\right)$, at a distance $d_{i}^{o}\left(t\right)$ from it. These two functions are given by:

(2) $$\begin{array}{l} \begin{array}{l} A\left(\phi_{i}\left(t\right),\psi_{i}^{g}\left(t\right)\right)=-k^{g}\left(\phi_{i}\left(t\right)-\psi_{i}^{g}\left(t\right)\right)\left(e^{-c_{1}d_{i}^{g}\left(t\right)}+c_{2}\right)\\ R\left(\phi_{i}\left(t\right),\psi_{i}^{o}\left(t\right)\right)=k^{o}\overset{N_{obst}}{\underset{n=1}{\sum }}r^{o_{n}}\left(\phi_{i}\left(t\right)\right) \end{array} \end{array}$$

The contributions of the individual obstacles to the repulsion function are given by:

(3) $$\begin{array}{l} r^{o_{n}}\left(\phi_{i}\left(t\right)\right)=\left(\phi_{i}\left(t\right)-\psi_{i}^{o_{n}}\left(t\right)\right)\left(e^{-c_{3}|\phi_{i}\left(t\right)-\psi_{i}^{o_{n}}\left(t\right)|}\right)\left(e^{-c_{4}d_{i}^{o_{n}}\left(t\right)}\right) \end{array}$$

In these equations, *k*^*j*^ and *c*_*j*_ are constants; *o*_*n*_ indicates the *nth* obstacle. Note that, in general, $\psi_{i}^{o_{n}}\left(t\right)$, which is the direction of the *nth* obstacle of the *ith* agent is time-dependent; for example, depending on the specific social interaction class it might be a function of the instantaneous heading direction of other agents.

The propagation speed dynamics are specified by the following stochastic differential equation:

(4) $$\begin{array}{l} \tau \dot{s_{i}}\left(t\right)=-s_{i}\left(t\right)+F_{i}\left(d_{i}^{g}\left(t\right)\right)+k_{i}^{\epsilon }\epsilon_{i}\left(t\right) \end{array}$$

where ϵ_*i*_(*t*) is Gaussian white noise. The nonlinear function *F*_*i*_ specifies how the agent's speed changes as a function of the distance from its goal:

(5) $$\begin{array}{l} F_{i}\left(d\right)=\frac{c_{5}}{1+e^{-c_{6}^{i}\left(d-c_{7}^{i}\right)}}-c_{8}^{i}e^{-k_{i}d}+c_{9}^{i} \end{array}$$

Critically, we choose this specific functional form because it provides us with enough flexibility to reproduce several relevant interaction classes, including the six fundamental interaction categories traditionally studied in psychophysical experiments (Blythe et al., 1999; Barrett et al., 2005; McAleer and Pollick, 2008): *Chasing, Fighting, Flirting, Following, Guarding*, and *Playing*.

**Figure 1:**
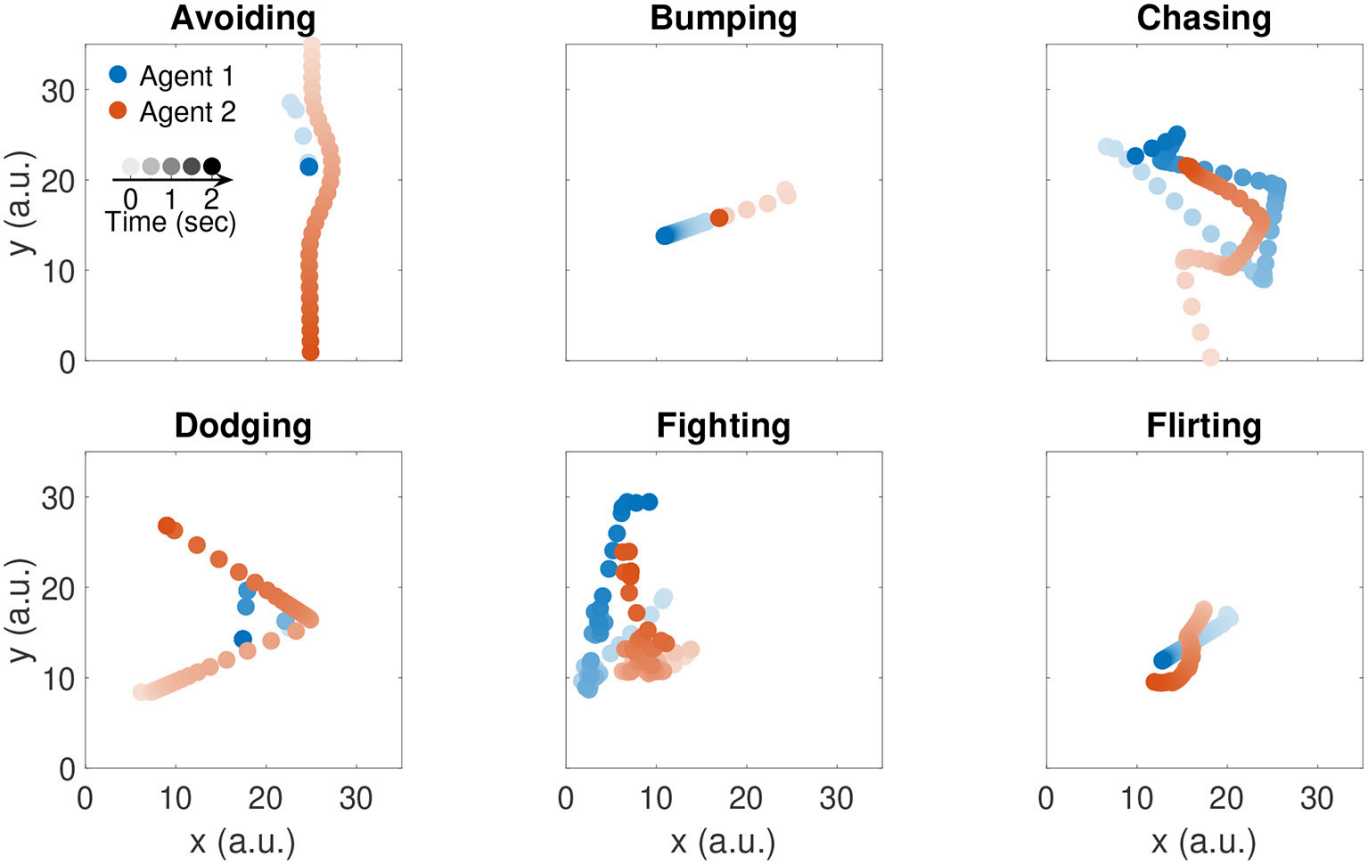
Trajectories of six example social interactions. Color indicates agent identity: agent 1 is represented in blue; agent 2 is represented in red. Color saturation indicates time: darker colors indicate recent time samples.

To generate the trajectories, we first randomly sample a series of goal points for the first agent from a two-dimensional uniform distribution over the 2D plane of action. Such goal points are commonly referred to as *via points*. We then use the instantaneous position of the first agent as goal position for the second agent. Samples that are too close to the current agent's position are rejected. Further details about the implementation of the generative model are provided in the Algorithm 1 box. Representative trajectories of six example social interactions are illustrated in Figure 1. Note that the speed control dynamics are not influenced by the presence of obstacles, since their effect was not needed to realistically capture the social interactive behaviors we chose to model.

**Algorithm 1:**
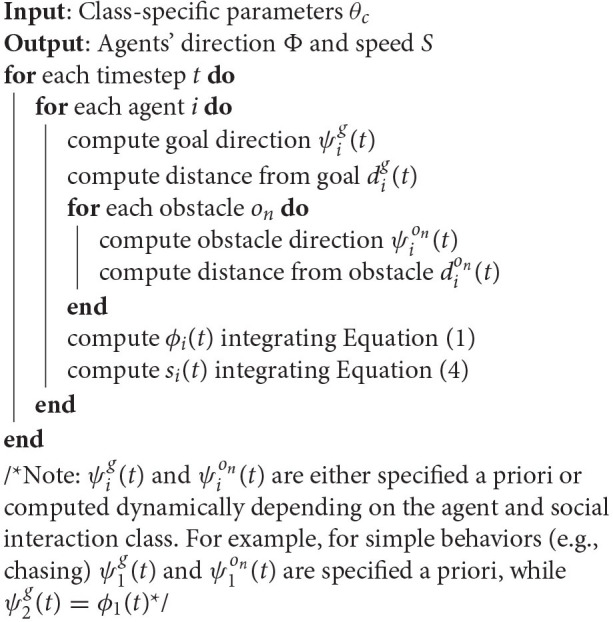
Pseudocode for trajectory generation

### 2.3. Model Validation

To assess whether our model is able to generate perceptually valid socially interactive behaviors, we carried out three behavioral experiments. In these experiments, we asked participants to categorize videos of interacting agents generated with our model in a free-choice task (Experiment 1), and in a forced-choice task (Experiment 2). Finally, we analyzed the semantic similarities between the labels chosen by the participants (Experiment 3).

#### 2.3.1. Dataset Generation

To validate our approach, we chose to model the six fundamental interaction classes (i.e., *Chasing, Fighting, Flirting, Following, Guarding*, and *Playing*; Blythe et al. 1999; Barrett et al. 2005; McAleer and Pollick 2008), and nine other relevant ones (i.e., *Avoiding, Bumping, Dodging, Frightening, Meeting, Pulling Pushing, Tug of War*, and *Walking*) resulting in a total of 15 interaction classes. To generate the trajectories corresponding to these classes, we simulated the model with 15 distinct parameter sets, which we identified through a simulation-based heuristic procedure. A list of the most critical parameters is presented in Table 1. The complete dataset we generated for our experiments included five random realizations of each interaction class, for a total of 75 videos. Each random realization is defined by different via points and noise realizations.

**Table 1:** Main model parameters.

**Interaction class**	**Agent 1**							**Agent 2**						
	***k***	***k*^**ϵ**^**	***c*_**5**_**	***c*_**6**_**	***c*_**7**_**	***c*_**8**_**	***c*_**9**_**	***k***	***k*^**ϵ**^**	***c*_**5**_**	***c*_**6**_**	***c*_**7**_**	***c*_**8**_**	***c*_**9**_**
Avoiding	0	0	1	1	5	3	0	0	0	0.4	1	0	2.7	0
Bumping	0	0.9	1	0.8	0	0	0	0	1	0.8	10	0	1	0
Chasing	0	0	1	10	7	0	0	0	0	1	1	7	0	0
Dodging	0	0	1	0.5	7	5	0	0	0	3	1	0	0	0
Fighting	0.1	0	1	1	3	1	0	0.1	1	1	1	3	1	0
Flirting	0	0	1	1	5	0	0	0.5	1	0.6	1	2	1	0
Following	0	0	1	10	7	0	0	0	0	1	4	4	0	0
Frightening	0	0	1	1	5	0	0	0	0	1	1	5	0	0.5
Guarding	0	0	1	1	5	0	0	0	0	1	1	3	0	0.5
Meeting	0	0.2	1	2	0	6	0	0.5	1	0.22	3	0	6	0
Playing	0	0	1	1	5	0	0	0	1	1	1	10	0	0.5
Pulling	0	0	1	10	0	2.6	0	0	0	0.9	5	0	2.6	0
Pushing	0	0	1	10	0	2.5	0	0	0	0.1	1	0	0	2.5
Tug of War	0	0.2	1	10	0	6	0	0	0.5	0.9	5	0	0	0.5
Walking	0	0.2	1	10	0	1	0	0	0	0.22	10	0	0	0

#### 2.3.2. Participants

A total of 39 participants with normal or corrected vision took part in the experiments: 13 in Experiment 1 (9 females, 4 males), ten in Experiment 2 (5 females, 5 males), and 16 in Experiment 3 (9 females, 7 males). All participants were college students attending the University of Tübingen and provided written informed consent before the experiments. All experiments were in full compliance with the Declaration of Helsinki. Participants were naïve to the purpose of the study and were financially compensated for their participation.

#### 2.3.3. Experiment Setup

In Experiment 1 and Experiment 2, participants sat in a dimly lit room in front of an LCD monitor (resolution: 1,920 × 1,080, refresh rate: 60*Hz*), at a distance of 60*cm* from it. To ensure that all participants would observe the stimuli with the same view parameters and the same distance from the screen, they were asked to place their heads in a chin-and-forehead rest during the experimental sessions. The experiments started with a short familiarization session during which the participants learned to use the computer interface. Subsequently, the participants were shown the videos generated with our model. Their task was to describe the videos by using their own words (Experiment 1) or by selecting labels among those provided to them (Experiment 2), and to provide animacy ratings through a standard 0–10 Likert scale. To increase the confidence in their answers, we gave participants the opportunity to re-watch each video up to three times. The videos were presented in pseudo-randomized order over five blocks. Five-minute rest breaks were given after each block. The animated videos always showed two agents moving in a 2D plane following speed and direction dynamics generated offline with our model. Critically, unlike in previous work (Blythe et al., 1999; Barrett et al., 2005), our agents were very simple geometrical shapes, namely a blue circle and a red rectangle (as in Tremoulet and Feldman, 2000); this choice ensured that participants' perception would not be biased by additional visual cues beyond the agents' motion and relative positions. In Experiment 3, subjects were asked to fill out a questionnaire to rate the semantic similarity between social interaction classes (0–10 Likert scale).

#### 2.3.4. Experiment 1

The first experiment was aimed at assessing whether subjects would perceive the motion of virtual agents generated with our model as a social interaction. The second goal of this experiment was the identification of unequivocal labels for the interaction classes generated with our model. To this end, we asked participants to watch all the videos in our stimulus set (section 2.3.1). After watching the videos, subjects were asked to provide their own interpretations by summarizing what they had perceived with a few sentences or keywords. Importantly, in this experiment, to make sure we would not bias the participants' perceptions, we did not provide them with any labels or other cues: they had to come up with their own words. In addition, subjects were asked to provide an animacy rating for each agent. The most commonly reported keywords were used as *ground-truth* interaction labels for the remaining experiments.

To test whether participants assigned different animacy ratings depending on agent identity and social interaction class, we fitted a linear mixed-effect model to the animacy ratings, with Agent and Social Interaction as fixed effects, and Subject as random effect:

(6) $$\begin{array}{l} {\text{Animacy}}_{sl}=\alpha_{0}+\overset{N_{a}}{\underset{i=1}{\sum }}\beta_{i}\cdot \text{Agent}\left(i,l\right)\\ \;+\overset{N_{c}}{\underset{i=1}{\sum }}\gamma_{i}\cdot \text{SocialInteraction}\left(i,l\right)+b_{0s}+\epsilon_{sl} \end{array}$$

In this model, Animacy_*sl*_ is the *lth* animacy rating reported by subject *s*, with *s* = 1, 2, ..., *N*_*s*_ and *l* = 1, 2, ..., *N*_*a*_*N*_*c*_; *N*_*a*_, *N*_*c*_, and *N*_*s*_ are the number of agents, social interaction classes, and subjects, respectively. Moreover, Agent(*i, l*) is a dummy variable that is equal to 1 when the rating *l* is for agent *i*, and 0 otherwise. Similarly, SocialInteraction(*i, l*) is a dummy variable that is equal to 1 when the rating *l* is for social interaction *i*, and 0 otherwise. Finally, *b*_0*s*_ is the subject-specific random effect [$b_{0s}\sim N\left(0,\sigma_{b}^{2}\right)$] and ϵ_*sl*_ are the residual error terms [$\epsilon_{sl}\sim N\left(0,\sigma^{2}\right)$]. Notably, the model was fitted with a sum-to-zero constrain, that is $\overset{N_{a}}{\underset{i=1}{\sum }}\beta_{i}=0$ and $\overset{N_{c}}{\underset{i=1}{\sum }}\gamma_{i}=0$; therefore, in this model, α_0_ represents the overall average animacy rating. All the analyses described in this and in the next sections were performed in MATLAB R2020a (The MathWorks, Natick, MA).

#### 2.3.5. Experiment 2

The second experiment was aimed at further studying the social interaction classes perceived by the participants while watching our animated videos. To this end, new subjects were exposed to a subset of the videos in our original dataset. Specifically, for this experiment we excluded the videos corresponding to the classes *Following, Guarding*, and *Playing*, as these tended either to be often confused with other classes, or to be labeled with a broad variety of related terms. Critically, unlike in Experiment 1, after watching the videos, participants were asked to describe the videos by choosing up to three labels, among those selected in Experiment 1.

To assess the classification performance, we computed the confusion matrix *M*. In this matrix, each element *m*_*i,j*_ is the number of times participants assigned the class *j* to a video from class *i*. Starting from *M*, we computed, for each social interaction class, Recall, Precision, and *F*_1_ score. Recall measures the fraction of videos of class *i* that are correctly classified, and is defined as $Recall_{i}=m_{i=j}/\overset{N_{c}}{\underset{j=1}{\sum }}m_{i,j}$. Precision measures the fraction of times participants correctly assigned the class *j* to a video, and is defined as $Precision_{j}=m_{i=j}/\overset{N_{c}}{\underset{i=1}{\sum }}m_{i,j}$. Finally, the *F*_1_ score is the harmonic mean of Precision and Recall; it measures the overall classification accuracy and is defined as *F*_1_ = 2·*Precision*·*Recall*/(*Precision* + *Recall*).

To evaluate whether some classes were more likely to be confused with each other, we computed, for each pair of classes (*i, j*), with *i* ≠ *j*, the empirical pairwise mislabeling probability, defined as $P_{MS}\left(i,j\right)=\left(m_{i,j}+m_{j,i}\right)/\left(\overset{N_{c}}{\underset{k=1}{\sum }}\underset{l\neq k}{\sum }m_{k,l}\right)$.

To assess whether participants improved their classification performance during the experiment, we computed the average Precision, Recall, and *F*_1_ score across social interaction class, as a function of experimental block; we then fitted linear models to test whether experimental block explained a significant fraction of variation in the performance measures defined above.

#### 2.3.6. Experiment 3

The third and last experiment was aimed at assessing whether there are interpretable semantic similarities among the labels provided in Experiment 2. Some interaction classes were misclassified by the participants in Experiment 2. This suggests that either the generated animated videos are not distinctive enough or that the classes semantically overlap with each other. To disambiguate between the two options, we ran a semantic survey test with a new set of participants. Participants in this experiment did not watch any video. After providing them with precise definitions for each social interaction class, we asked them to indicate the level of semantic similarity for each pair of classes, by providing rates ranging from 0 to 10. Specifically, using this scoring system, participants were asked to assign 0 to pairs of classes perceived as not sharing any semantic similarity, and 10 to those perceived as equivalent classes.

To assess the geometry of the semantic similarity space, we first transformed all the similarity ratings *s* into distance ratings *d* by computing their complement (i.e., *d* = 10 − *s*), and then rescaled them between 0 and 1. All the resulting semantic distances collected from participant *i* were then stored in a matrix *D*^*i*^. In this matrix, $D_{j,k}^{i}=0$ if the classes *j* and *k* were considered as semantically equivalent by subject *i*; $D_{j,k}^{i}=1$ if the classes *j* and *k* were considered as semantically unrelated. We then used non-metric multidimensional scaling (MDS; Shepard, 1962a,b) to visualize in a 2D space the underlying relational structure contained in the distance matrix.

To determine whether some groups of classes were consistently considered as semantically similar, we performed agglomerative hierarchical clustering on the distance matrix *D* using the Ward's linkage method (Ward, 1963), which minimizes the within-cluster variance. Clusters were then identified using a simple cut-off method, using as a threshold τ = 0.7 · *M*_*WD*_, where *M*_*WD*_ is the maximum observed Ward's distance.

Finally, to estimate whether the semantic similarity between pairs of classes explained the mislabelings observed in Experiment 2, we computed the Pearson's correlation coefficient (ρ) between the empirical mislabeling probability *P*_*MS*_(*j, k*) measured in Experiment 2 and the semantic distance *D*(*j, k*).

## 3. Results

### 3.1. Experiment 1

**Figure 2:**
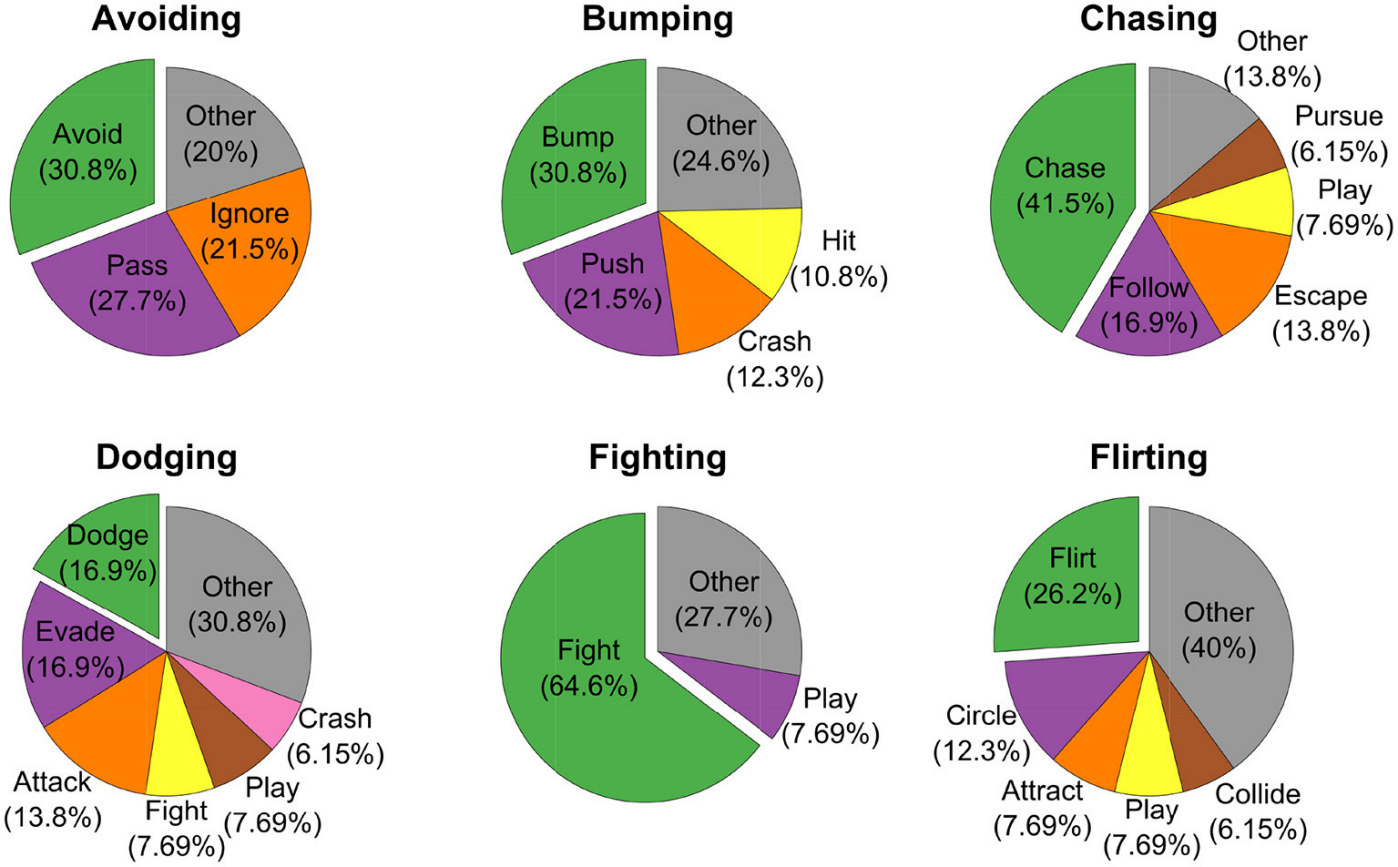
Distribution of reported keywords for six example social interactions. Pie charts' titles indicate the true classes. Individual slices are assigned to all the keywords reported in Experiment 1 occurring with a frequency >5%. Keywords reported with a frequency <5% are pooled together in the slice *Other* (in gray). Offset slices (in green) represent the most frequently reported keywords.

As mentioned above, participants in this experiment were completely free to provide interpretations about the videos through either labels or short sentences. For each video class, we pooled together all the definitions and labels, and we considered the most used term as the *ground-truth* class label. Figure 2 summarizes the reported labels for six example social interaction classes. The pie charts show that some classes such as *Avoiding* and *Fighting* tended to be consistently described with very few labels (i.e., 2 − 3). Other classes such as *Dodging* were instead described with more labels (i.e., 6). Regardless of the number of labels used to describe a social interaction class, these were generally semantically similar. For example, some classes were named interchangeably depending on the perspective from which subjects reported their interpretation about the videos. A typical example of this issue is the ambiguity between the classes *Pulling* and *Pushing*. On the other hand, some other classes (for instance *Bumping* and *Pushing*) were sometimes misclassified regardless of the perspective from which subjects might have observed the videos.

**Figure 3:**
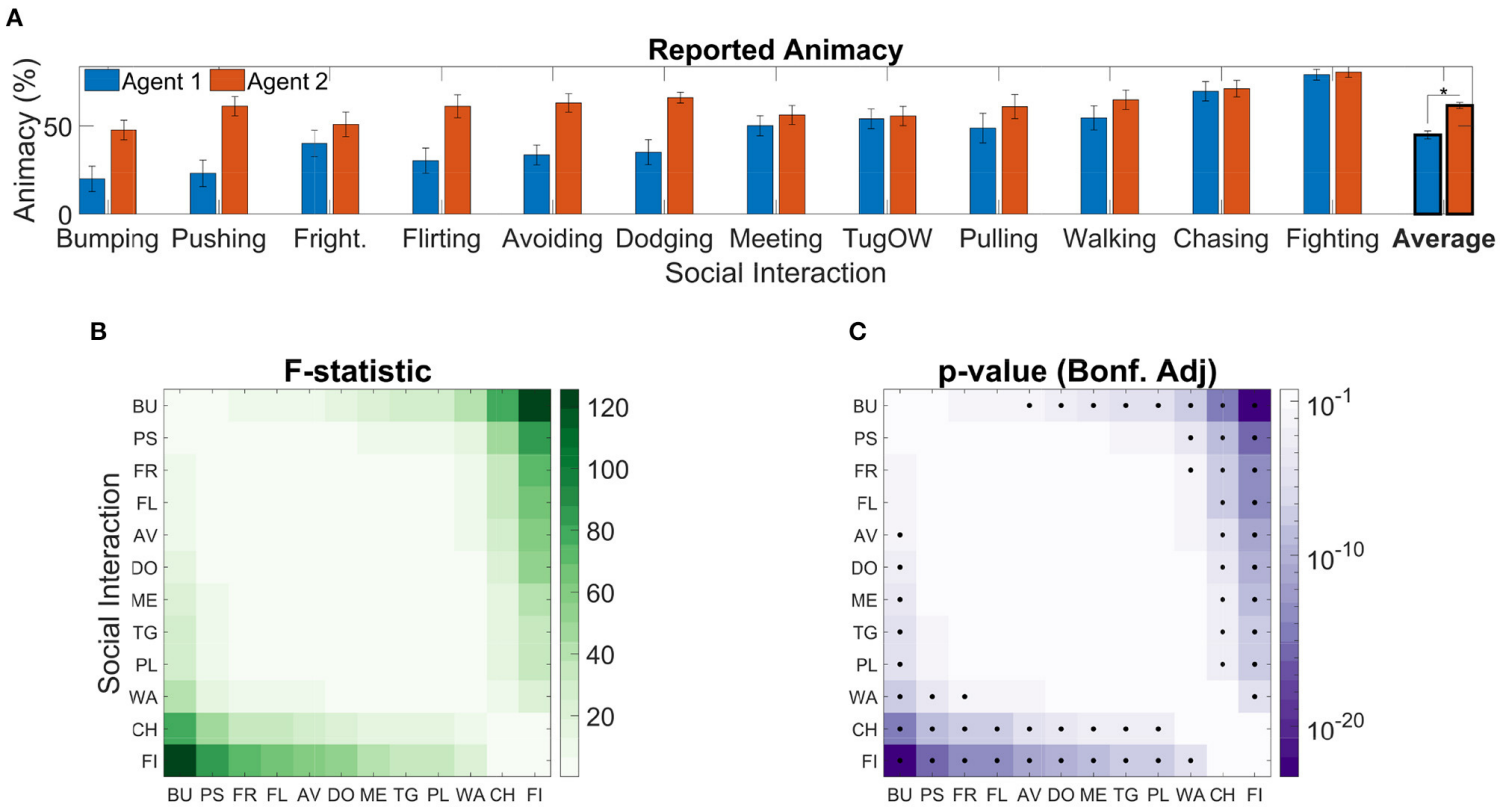
Reported agent animacy. **(A)** Mean animacy ratings obtained in Experiment 1; error bars represent standard errors; results are rescaled between 0 and 100. Classes are sorted in ascending order by average across-agent animacy rating. The asterisk denotes a significant effect (*p* < 0.05) of Agent on Animacy [*F*_(1,299)_ = 99.98, *p* = 1.74 · 10^−20^]. **(B)** F-statistics of *post-hoc* tests to assess the difference in animacy ratings between social interaction classes [i.e., *F*_(1,299)_]. **(C)** Bonferroni adjusted *p*-values corresponding to the F-statistics reported in **(B)**; black dots represent significant pairwise differences.

Average animacy ratings are reported in Figure 3A, with classes sorted in ascending order of average across-agent animacy. Agents, were consistently perceived as animate [α_0_ = 53.27%, *t*_(299)_ = 11.72, *p* = 2.3·10^−26^]. This is consistent with the fact that self-propulsion (Csibra, 2008), goal directedness (van Buren et al., 2016), being reactive to social contingencies (Dittrich and Lea, 1994), acceleration (Tremoulet and Feldman, 2000), and speed (Szego and Rutherford, 2008) are the most prominent cues for perceived animacy in psychophysical experiments. Moreover, the blue circle was consistently rated as less animate than the red rectangle [β_1_ = −β_2_ = −8.37%, *t*_(299)_ = −10, *p* = 1.74 · 10^−20^], consistently with the finding that geometrical figures with a body axis are perceived as more animate than those without one, such as circles (Tremoulet and Feldman, 2000).

We further found a significant effect of social interaction on animacy [*F*_(11,299)_ = 18.3, *p* = 8.29 · 10^−28^]; this suggests that certain classes of social interactions tended to elicit stronger animacy percepts than others. To assess which specific pairs of classes were assigned significantly different animacy rating, we performed *post-hoc F*-tests. This analysis revealed that some classes consistently received higher average animacy ratings: for example, *Fighting* received higher animacy ratings than all other classes [*F*_(1,299)_ ≥ 24.04, $p_{adj}\leq 1.03\cdot 10^{-4}$], with the exception of *Chasing*, which was rated similarly [*F*_(1,299)_ = 5.25, *p*_*adj*_ = 1]. Analogously, *Bumping* tended to receive lower animacy ratings than all other classes [*F*_(1,299)_ ≥ 12.44, *p*_*adj*_ ≤ 0.03], with the exception of *Pushing, Frightening*, and *Flirting*, which were rated similarly [*F*_(1,299)_ = 8.42, *p*_*adj*_ ≥ 0.26]. We report in Figure 3B all the *post-hoc* F-statistics, and in Figure 3C all the corresponding Bonferroni adjusted *p*-values.

### 3.2. Experiment 2

**Figure 4:**
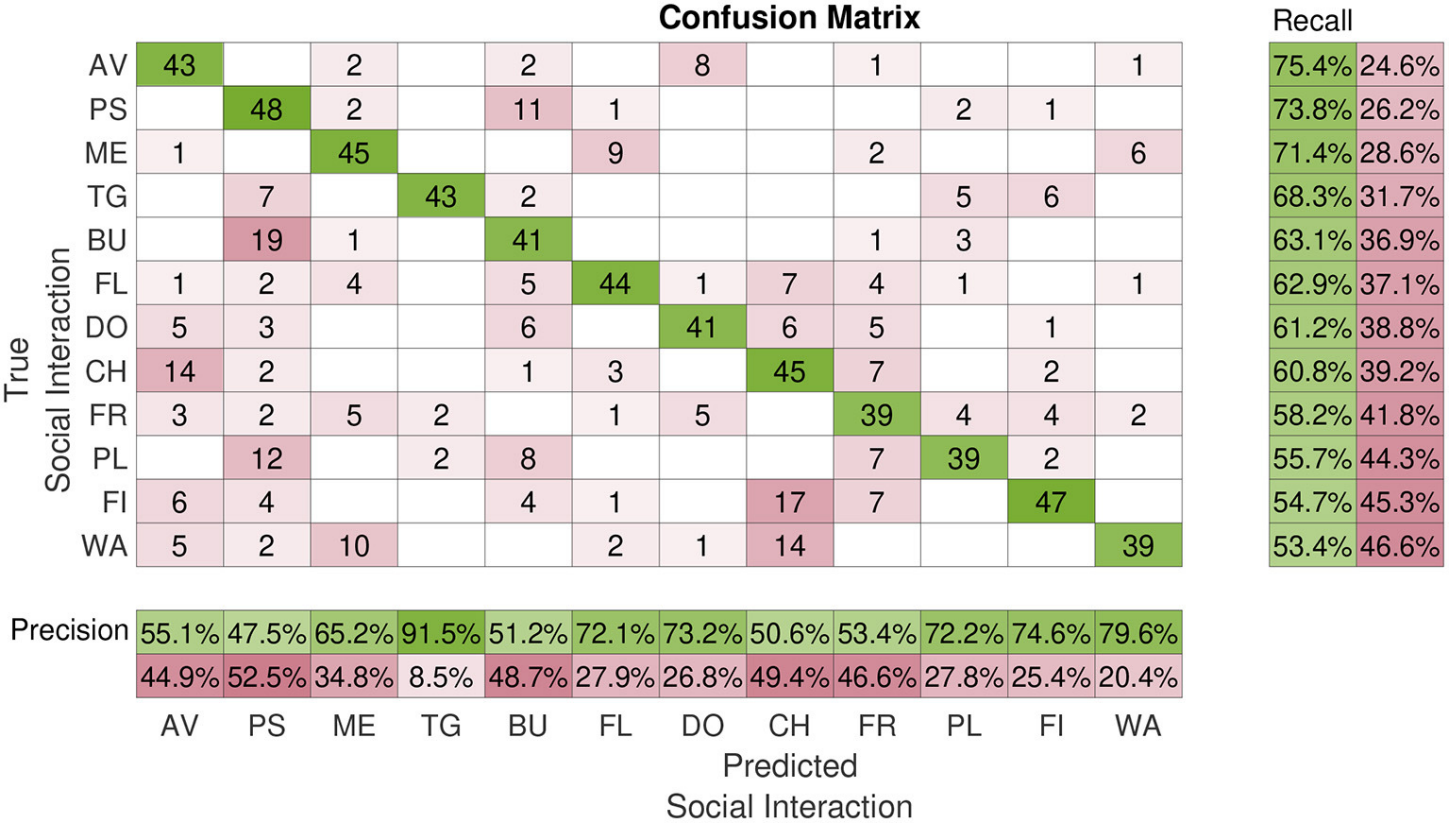
Average classification performance. This figure shows the confusion matrix of the classification experiment (Experiment 2). Rows represent the true interaction class; columns the interaction class reported by the participants in Experiment 2. Matrix entries *m*_*i,j*_ report the number of times participants assigned the class *j* to a video from class *i*. Rows and columns are sorted by decreasing Recall. AV, avoiding; BU, bumping; CH, chasing; DO, dodging; FI, fighting; FL, flirting; FR, frightening; ME, meeting; PL, pulling; PS, pushing; TG, tug of war; WA, walking.

Figure 4 shows the total confusion matrix *M* of the classification task. Rows and columns are sorted by decreasing Recall. *Avoiding* was the most accurately classified class by our participants (*Recall* = 75.4%). However, even the hardest class was classified with largely above-chance accuracy (*Walking*: *Recall* = 53.4%; chance level: 8.3%). Nonetheless, there are obviously some misclassifications, especially between *Bumping* and *Pushing* (*m*_*BU,PS*_ = 19, *m*_*PS,BU*_ = 11), and between *Fighting* and *Chasing* (*m*_*FI,CH*_ = 17, *m*_*CH,FI*_ = 2). These two kinds of mislabeling alone accounted for a large fraction of the total number of mislabelings [*P*_*MS*_(*BU, PS*) = 9.8%, *P*_*MS*_(*FI, CH*) = 6.2%].

**Figure 5:**
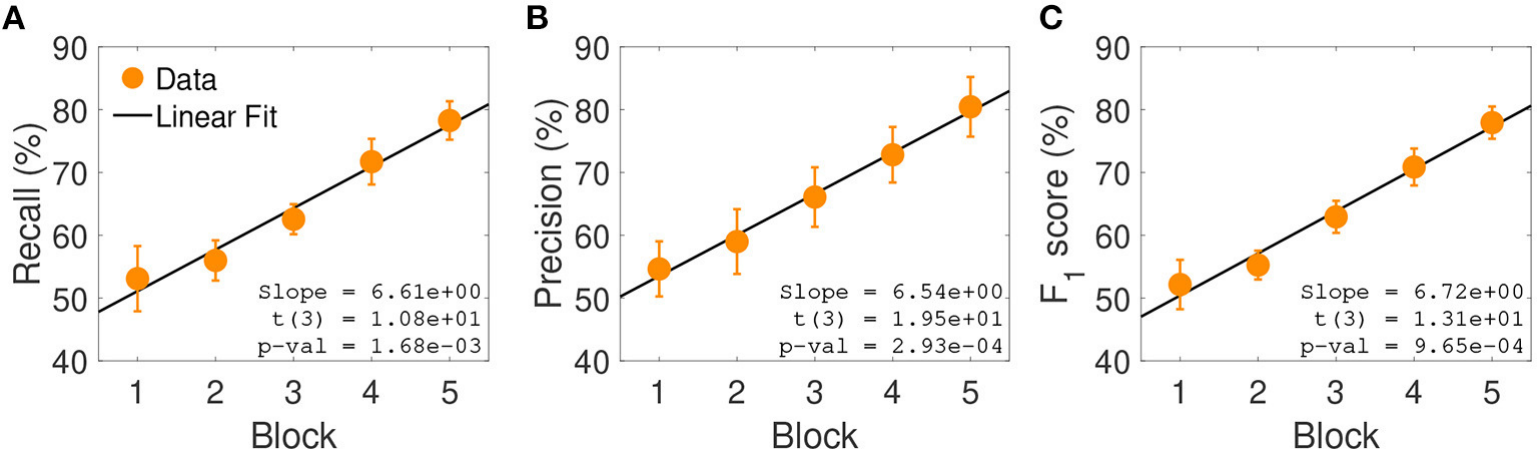
Classification performance across experimental blocks. **(A)** Average block-wise recall. Results are averaged across subjects and social interactions; error bars represent standard errors. Insets show the slope of the estimated linear model, the corresponding *t*-statistic and *p*-value. **(B)** Average block-wise precision. **(C)** Average block-wise *F*_1_ score.

One possible reason for this misclassification could be the fact that these labels are semantically intrinsically similar and even real videos of these types of social interactions could be mislabeled. This line of reasoning is supported by the fact that in Experiment 1, *Pushing* was the second preferred keyword used to label videos of class *Bumping* (see Figure 2). Interestingly, both Precision and Recall (and thus *F*_1_ score) significantly improved across experimental blocks [Precision: *t*_(3)_ = 19.5, *p* = 2.93 · 10^−4^; Recall: *t*_(3)_ = 10.8, *p* = 1.68 · 10^−3^; see Figure 5]. This indicates a latent learning of the categorization of the classes, which is remarkable since no external feedback about the correctness of the class assignments was provided during the experiment. Such a learning was particularly evident for the following often-confused pairs: *Tug of War* vs. *Pulling, Frightening* vs. *Avoiding*, and *Fighting* vs. *Pushing* (not shown).

### 3.3. Experiment 3

**Figure 6:**
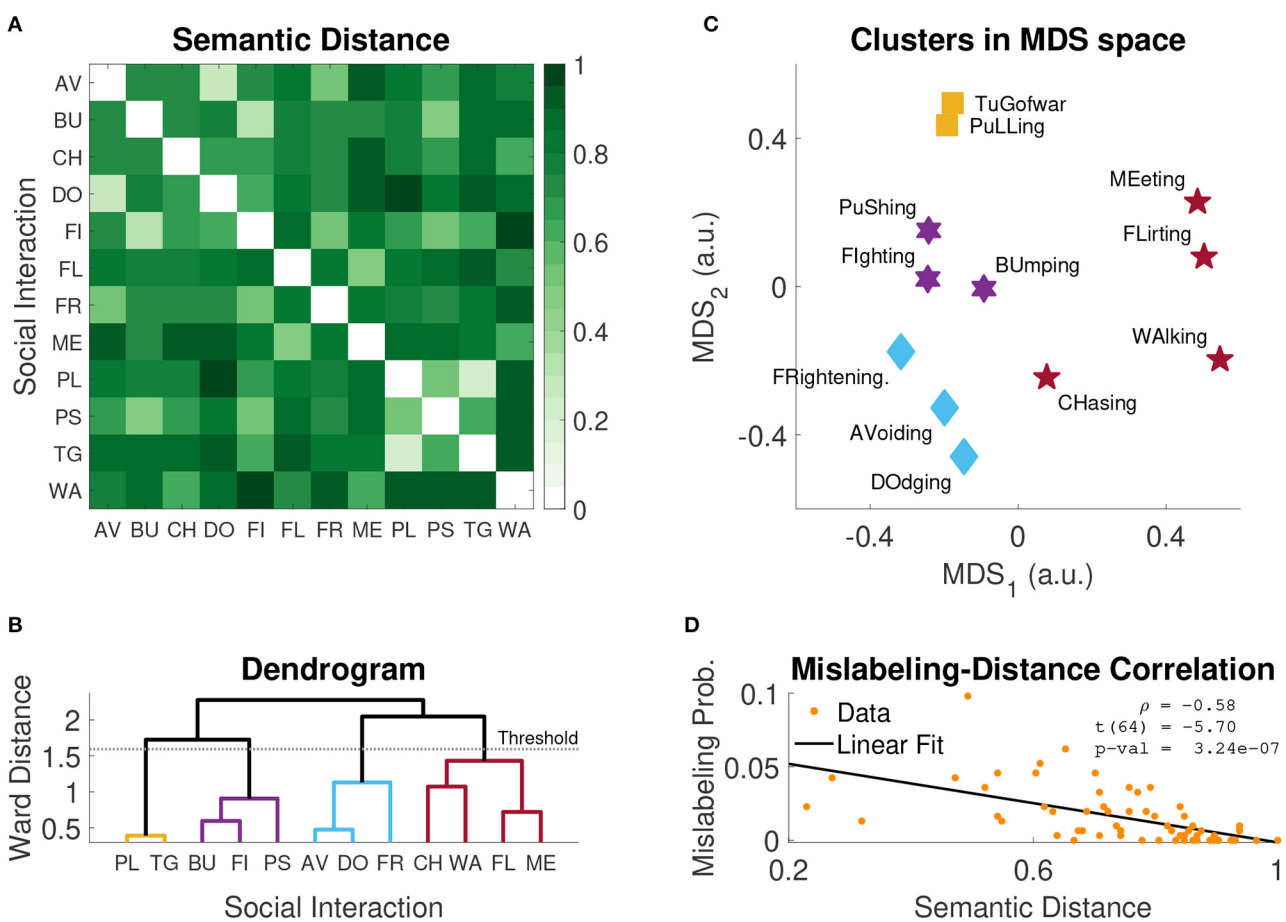
Cluster analysis results. **(A)** Average semantic distances obtained in Experiment 3. **(B)** Dendrogram of hierachical clustering; the horizontal line represents the cut-off threshold used to identify the clusters (i.e., 0.7*M_*WD*_, where *M*_*WD*_ is the maximum Ward distance). **(C)** Clusters of social interactions plotted in low dimensional distance-preserving 2D space identified with Multidimensional Scaling (MDS). **(D)** Average mislabeling probability (Experiment 2) as a function of semantic distance (Experiment 3); inset reports the Pearson's correlation coefficient, the corresponding *t*-statistic and *p*-value. AV, avoiding; BU, bumping; CH, chasing; DO, dodging; FI, fighting; FL, flirting; FR, frightening; ME, meeting; PL, pulling; PS, pushing; TG, tug of war; WA, walking.

The pairwise semantic distance matrix *D* is plotted in Figure 6A: light shades of green indicate semantically close social interaction classes, while darker shades indicate semantically distant classes. The two pairs associated with the highest mislabeling probability in Experiment 2, *Bumping*-*Pushing*, and *Fighting*-*Chasing* [*P*_*MS*_(*BU, PS*) = 9.8%, *P*_*MS*_(*FI, CH*) = 6.2%] were generally considered as semantically similar [*d*(*BU, PU*) = 0.49, *d*(*FI, CH*) = 0.65]; however, they were not the most similar pairs. Rather, the three most semantically similar pairs were *Pulling*-*Tug of War, Avoiding*-*Dodging*, and *Bumping*-*Fighting* [*d*(*PU, TG*) = 0.23, *d*(*AV, DO*) = 0.27, *d*(*BU, FI*) = 0.32]. Nevertheless, regardless of this apparent discrepancy for these few extreme examples, mislabeling probability *P*_*MS*_(*i, j*) and semantic distance *d*(*i, j*) were significantly anti-correlated [ρ = −0.58, *t*_(64)_ = −5.7, *p* = 3.24 · 10^−7^; Figure 6D]; this suggests that the more semantically similar two social interaction classes are, the more likely they are of being confused in a video labeling task.

Multidimensional scaling (MDS) provides a compact 2D visualization of the semantic similarity space (Figure 6C). Since MDS is inherently spatial, items that were rated as being highly similar are spatially close to each other in the final map. The map effectively shows which classes of social interactions are semantically similar and which are not. For example, let us consider the hypothetical groups *G*_1_ ={Tug of War, Pulling} and *G*_2_ ={Frightening, Avoiding, Dodging}. Participants recognized that *Tug of War* and *Pulling* involve similar interactions between the agents, and that these interactions are different from those occurring in the classes *Frightening, Avoiding*, and *Dodging*. For this reason, participants tended to assign high pairwise similarity scores to intra-group pairs, and low to inter-group pairs. This pattern of scoring is captured by MDS and evident in the resulting map (Figure 6C).

The agglomerative hierarchical cluster analysis on the distance matrix *D* (Figure 6B) confirms this intuition and identifies four distinct semantic clusters; such clusters are visualized in the MDS map with four different symbols (Figure 6C). This analysis supports the conclusion that misclassified labels tend to belong to the same semantic cluster. While not all misclassifications can be explained by semantic similarity, many confusions can be accounted for by this factor. For example, *Pushing* vs. *Bumping, Walking* vs. *Meeting, Avoiding* vs. *Dodging*.

To summarize, our analysis of semantic similarity shows that many of the labeling confusions observed in Experiment 2 can be explained by the semantic similarity of the class labels.

## 4. Discussion

In this work, we introduced a novel framework for the automatic generation of videos of socially interacting virtual agents. The underlying model is a nonlinear dynamical system that specifies heading direction and forward speed of the agents. Our model is able to generate as many as 15 different interaction classes, defined by different parameter sets. We validated our model with three different behavioral experiments, in which participants were able to consistently identify the intended interaction classes. Our model is thus suitable for the automatic generation of animations of socially interacting agents. Furthermore, the generation process is also amenable to full parametric control. This feature allows the creation of highly-controlled and arbitrarily-large datasets for in-depth psychophysical and electrophysiological characterization of the perception of social interactions. The model thus overcomes the major limitations that come with hand-crafted, hard-coded, rule-based, and real-video-based approaches (1) to visual stimuli generation. Importantly, the generative nature of the model, makes it a valuable tool also for the development of mechanistic and neural *decoder* models of social perception: model responses to the heterogeneous set of highly-controlled social stimuli here introduced can be rigorously tested for the development of more accurate and brain-like decoder models that replicate human behavioral and neural responses. Recent work (Shu et al., 2018, 2019, 2020), aimed at building a mechanistic model of social inference, used a similar approach.

Shu et al. (2019, 2020) also proposed generative models of social interactions. Unlike the ones proposed in these studies, the generative model introduced in this work does not directly lend itself to the study of the interactions between intuitive physics and social inferences (Battaglia et al., 2013). However, substantial evidence suggests that physical and social judgments are mediated by different brain regions (Isik et al., 2017; Sliwa and Freiwald, 2017). More importantly, our model is not limited to describing cooperative and obstructive behaviors and thus seems better suited to study more general social interaction classes.

The identification of suitable parameters for the classes modeled in this work was not automatic: it was conducted using a simulation-based heuristic procedure. This is an obvious limitation of our work. Nevertheless, once the parameters are available, they can be used to automatically generate arbitrary numbers of coupled trajectories for each interaction class (by randomly sampling initial conditions, via-points, and noise). With this procedure, we were able to find suitable parameters for only 15 specific interaction classes. However, to the best of our knowledge, no other method is able to automatically generate more than a handful of individual or socially-interactive behaviors (Blackwell, 1997; Paris et al., 2007; Luo et al., 2008; Russell et al., 2017; Shu et al., 2019, 2020). Future work will extend the range of modeled classes by using system identification methods (e.g., Schön et al., 2011; Gao et al., 2018; Gonçalves et al., 2020) to automatically extract model parameters from preexisting trajectories—extracted, for example, from real videos.

Another possible limitation of our work is that all our participants were recruited from a German university; while this might, in theory, represent a biased sample, previous studies (Rimé et al., 1985) suggest that the perception of social interactions from impoverished stimuli is a phenomenon that is highly stable across cultures. Specifically, these authors showed that African, European, and Northern American participants provided similar interpretations to animated videos of geometrical shapes. This suggests that our findings would not have significantly changed if we had recruited a more heterogeneous sample.

In this work, we used the trajectories generated by our model to animate simple geometrical figures. The resulting abstract visual stimuli can be directly applied to characterize the kinematic features underlying the inference of social interactions. However, the trajectories can also be used as a basis for richer visual stimuli. For example, in ongoing work, we have been developing methods to link the speed and direction dynamics generated by the model to articulating movements of three-dimensional animal models. This approach allows the generation of highly controlled and realistic videos of interacting animals, which can be used to study social interaction perception in the corresponding animal models with ecologically valid stimuli. Furthermore, contrasting the neural responses to impoverished and realistic visual stimuli can help identify the brain regions and neural computations mediating the extraction of the relevant kinematic features and the subsequent construction of social percepts.

Finally, even though the proposed model is mainly aimed to provide a tool to facilitate the design of in-depth psychophysical and electrophysiological studies of social interaction perception, we speculate that it can also be helpful in the development of machine vision systems for the automatic detection of social interactions. Specifically, the development of effective modern machine vision systems tends to be heavily dependent on the availability of large numbers of appropriately-labeled videos of social interactions (Rodŕıguez-Moreno et al., 2019; Stergiou and Poppe, 2019). A popular approach to this problem is to use clips extracted from already existing (YouTube) videos and movies. However, one of the reasons why feature-based (e.g. Kumar and John, 2016; Sehgal, 2018) and especially deep-neural-network-based (e.g., Karpathy et al., 2014; Carreira and Zisserman, 2017; Gupta et al., 2018) vision systems require *big data* is that they need to learn to ignore irrelevant information that is inevitably present in real videos. Therefore, we hypothesize that pre-training such systems with stylized videos of socially interacting agents—such as the very same generated by our model or appropriate avatar-based extensions—might greatly reduce their training time and possibly improve their performance. Future work will test this hypothesis.

To sum up, this work introduced a novel generative model of social interactions. The results of our psychophysical experiments suggest that the model is suitable for the automatic generation of arbitrarily-numerous and highly-controlled videos of socially interacting agents for comprehensive studies of animacy and social interaction perception. Our model can also be potentially used to create large, noise-free, and annotated datasets that can facilitate the development of mechanistic and neural models of social perception, as well as the design of machine vision systems for the automatic recognition of human interactions.

## Data Availability Statement

The original contributions presented in the study are included in the article/Supplementary Material, further inquiries can be directed to the corresponding author/s.

## Ethics Statement

The studies involving human participants were reviewed and approved by the ethics board of the University of Tübingen. The patients/participants provided their written informed consent to participate in this study.

## Author Contributions

All authors listed have made a substantial, direct and intellectual contribution to the work, and approved it for publication.

## Conflict of Interest

The authors declare that the research was conducted in the absence of any commercial or financial relationships that could be construed as a potential conflict of interest.
